# Intratumor Heterogeneity and Treatment Resistance of Solid Tumors with a Focus on Polyploid/Senescent Giant Cancer Cells (PGCCs)

**DOI:** 10.3390/ijms241411534

**Published:** 2023-07-16

**Authors:** Razmik Mirzayans, David Murray

**Affiliations:** Department of Oncology, Cross Cancer Institute, University of Alberta, Edmonton, AB T6G 1Z2, Canada; david.murray5@ahs.ca

**Keywords:** cancer therapy, intratumor heterogeneity, polyploid giant cancer cells, senescence, apoptosis, anastasis, preclinical assays, precision oncology

## Abstract

Single cell biology has revealed that solid tumors and tumor-derived cell lines typically contain subpopulations of cancer cells that are readily distinguishable from the bulk of cancer cells by virtue of their enormous size. Such cells with a highly enlarged nucleus, multiple nuclei, and/or multiple micronuclei are often referred to as polyploid giant cancer cells (PGCCs), and may exhibit features of senescence. PGCCs may enter a dormant phase (active sleep) after they are formed, but a subset remain viable, secrete growth promoting factors, and can give rise to therapy resistant and tumor repopulating progeny. Here we will briefly discuss the prevalence and prognostic value of PGCCs across different cancer types, the current understanding of the mechanisms of their formation and fate, and possible reasons why these tumor repopulating “monsters” continue to be ignored in most cancer therapy-related preclinical studies. In addition to PGCCs, other subpopulations of cancer cells within a solid tumor (such as oncogenic caspase 3-activated cancer cells and drug-tolerant persister cancer cells) can also contribute to therapy resistance and pose major challenges to the delivery of cancer therapy.

## 1. Introduction

**Figure 1:**
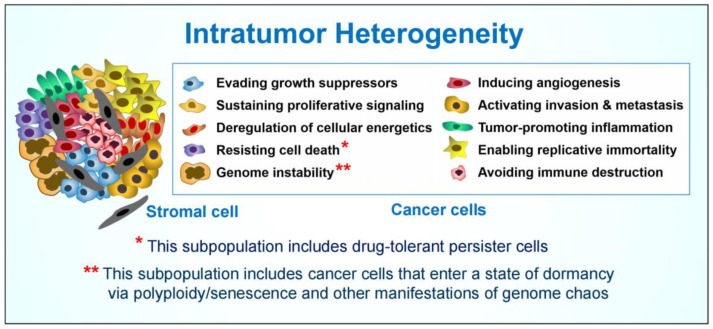
Complex heterogeneity within an individual solid tumor (adapted from [8]).

The discovery of the DNA damage surveillance network (also called the DNA damage response) in the 1990’s led to a model in which p53 and other key players in this network either activate cell cycle checkpoints following anticancer treatment to facilitate the repair of genomic injury and promote cell survival or eliminate injured cells from the proliferating population via apoptosis and other genetically-controlled (regulated) cell death pathways (e.g., [1,2,3,4]). This model is still being widely cited and the concepts embodied therein have been key drivers of innovations in cancer research. In the past decade, however, our understanding of the complexity of cancer cell responses to therapeutic agents has grown far beyond this canonical model of repair and survive or die through regulated cell death pathways. To this end, single cell biology has revealed that different subpopulations of cancer cells within a solid tumor/tumor-derived cell line can exhibit therapy resistance via different molecular and cellular processes (Figure 1), a phenomenon referred to as intratumor heterogeneity [5,6,7,8,9,10,11,12].

A Perspective article has been recently published in *Nature Cancer* [13] which has focused on two therapy resistant cancer subpopulations: drug-tolerant persister cells (characterized as cancer cells without resistance-associated mutations that nonetheless survive treatment) and, paradoxically, cancer cells undergoing apoptosis and other modes of regulated cell death (e.g., necroptosis, ferroptosis, and pyroptosis).

In this review, we discuss the importance of another subpopulation of cancer cells that contributes to therapy resistance and disease recurrence: dormant (proliferation arrested) cancer cells exhibiting various manifestations of genome chaos (polyploidy, multinucleation, micronucleation, and/or senescence) (Section 2). In addition, we discuss important considerations when assessing cancer cell radiosensitivity and chemosensitivity, with the purpose of clarifying the biological consequence(s) that the term “sensitivity” refers to (Section 3 and Section 4), and possible reasons why the impact of cancer cell dormancy on disease recurrence continues to be overlooked in most cancer therapy-related preclinical studies (Section 5).

Terminology Clarification: The Nomenclature Committee on Cell Death (NCCD) [14] and others (e.g., [15,16]) have published cautionary articles formulating several caveats concerning the misuse of terminology (e.g., cell survival, apoptosis, necrosis, autophagy, viability) and concepts that have slowed down progress in the area of cell death research. In their 2009 article [14], the NCCD stated that “…cells that are arrested in the cell cycle (as it occurs during senescence) should be considered as alive, and the expression ‘replicative cell death’ (which alludes to the loss of clonogenic potential), as it is frequently used by radiobiologists, should be abandoned.” Our discussion below will be in keeping with the NCCD recommendations.

Please note: We have used quotation marks for the term “lethality” throughout this review because the biological assessment of cancer cell death is largely inferred by the use of preclinical assays that do not distinguish dead cancer cells and dying cancer cells that have the potential to recover and generate aggressive variants [10,13,16]. In addition, although host immune response is crucial in the outcome of cancer therapy, as recently discussed by us [17] and others (e.g., [18,19]), the main focus of the current review is on responses measured in preclinical cell-based studies.

## 2. Therapy-Induced Cancer Cell Polyploidy/Senescence and Disease Recurrence

Solid tumors are complex systems that contain heterogeneous cancer cells with remarkably different sizes and genomic contents (reviewed in [20]). These encompass giant cells with a highly enlarged nucleus, multiple nuclei, and or multiple micronuclei. A subset of giant cells also exhibit senescence-like features, such as expression of the senescence marker p21^WAF1^ (p21) and positive staining in the senescence-associated β-galactosidase (SA β-Gal) assay ([20,21,22,23,24,25,26]. We will refer to these giant cells (with or without senescence features) as polyploid/senescent giant cancer cells (PGCCs). Although PGCCs constitute only a subset of cells within a solid tumor/tumor-derived cell line, their frequency can increase markedly under hypoxia or following treatment with genotoxic and non-genotoxic anticancer agents [17]. PGCCs represent a numerical “chaotic genome” subtype [27,28] and exhibit the potential to promote tumor repopulation and metastasis that can ultimately kill the patient (reviewed in [29,30,31]).

**Figure 2:**
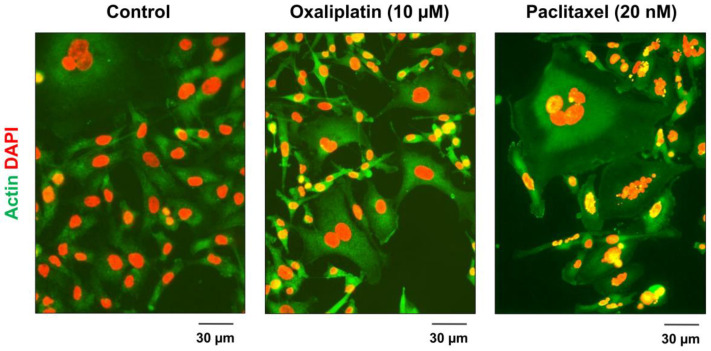
Fluorescence images showing the morphology of MDA-MB-231 cells before (control) and after treatment with the indicated drugs for 3 days. Reproduced from Mirzayans et al. [32].

Representative images obtained by us [32] for the MDA-MB-231 breast cancer cell line before and after exposure to chemotherapeutic drugs are presented in Figure 2 to illustrate the degree of treatment-response heterogeneity that can occur within a given cancer cell line.

### 2.1. Prevalence and Prognostic Value of PGCCs

In 2014, Coward and Harding published an article in *Frontiers in Oncology* entitled “Size does matter: why polyploid tumor cells are critical drug targets in the war on cancer” [23]. These authors provided a clear definition of different degrees of ploidy (polyploidy, tetraploidy, aneuploidy, hyperdiploidy) and reviewed the literature involving preclinical [21,33] as well as clinical [22,33] studies demonstrating the role of giant polyploid cells in therapy resistance and tumor repopulation after therapy. These authors also reported data, obtained by the application of an improved flow cytometry method [34], demonstrating that the prevalence of PGCCs in patient tumors may be higher than is generally appreciated. The study involved low-passage primary cell lines derived from ten glioblastoma patients; the cells were maintained using the method developed by Lee et al. [35], which permits in vitro propagation of glioblastoma cells under conditions that closely mimic the genotype, gene expression profile, and biology of their parental primary tumors. The lowest frequency of polyploid cells in tumor samples was 1 in 20 cells (i.e., 5% of total cells), leading the authors to speculate that brain tumors with volumes of ~1 cm^3^ may contain at least five million polyploid cells.

Recently, Trabzonlu, Pienta, Amend, and coworkers [36] have highlighted numerous studies reported since 2013 demonstrating that the presence of giant cancer cells in the polyploidy/senescence state (called PACCS, for polyaneuploid cancer cells, by these authors) is associated with worse prognosis, higher tumor grade, poor differentiation, and/or advanced disease stage across different solid tumor types. These include glioma, anorectal melanoma, laryngeal cancer, breast cancer, ovarian cancer, colon cancer, and prostate cancer ([37,38,39,40,41,42,43,44,45,46]; see Table 1 for details). These authors [36] also reported the results of tissue microarrays that were prepared from formalin-fixed, paraffin-embedded blocks of normal/benign and prostate cancer specimens. The purpose of this study was to systematically assess the presence and importance of PGCCs in prostate cancer patients who underwent radical prostatectomy with curative intent to treat their presumed localized tumor. The results identified PGCCs as significant prognostic factors for metastasis in these patients.

### 2.2. Formation and Fate of PGCCs

**Figure 3:**
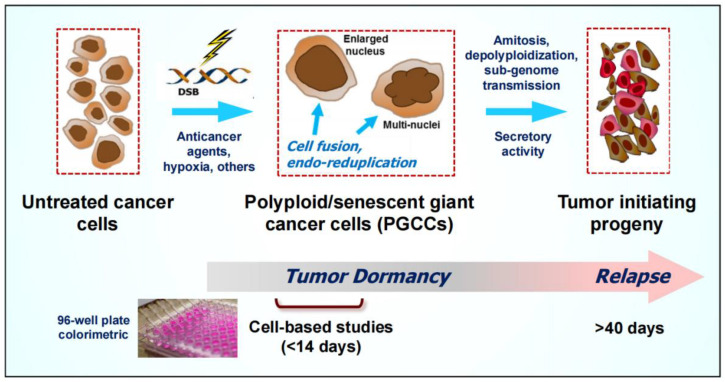
Cartoon illustrating the generation and fate of polyploid/senescent giant cancer cells (PGCCs). Anticancer treatment triggers the creation of PGCCs that often enter a state of dormancy (active sleep) and thus might be overlooked or scored as “dead” in conventional preclinical assays. A subset of PGCCs, however, remain viable, secrete growth promoting factors, and can give rise to therapy resistant and tumor repopulating progeny through neosis (nuclear budding and bursting), depolyploidization involving meiosis and self-renewal genes, and sub-genome transmission (transfer of nuclear material into surrounding cells via cytoplasmic tunnels). For further details, see [20].

The mechanisms that lead to the generation of PGCCs and their fate have been well documented and extensively reviewed (see, e.g., the Editorial in the recent special issue on PGCCs in *Seminars in Cancer Biology* [30]; also see Figure 3). In short, under stressful conditions, cancer cells within a solid tumor/tumor cell line undergo a complex series of adaptations, including endoreduplication and cell fusion, that result in the development of PGCCs that often enter a state of dormancy (durable proliferation arrest). These giant cells may contribute to tumor repopulation following cancer therapy by at least four mechanisms: (i) depolyploidization through undergoing a complex genome reduction process, mediated by key regulators of mitosis, meiosis and self-renewal, ultimately resulting in the emergence of para-diploid progeny (i.e., containing a near-diploid number of chromosomes) that exhibit recovery of proliferative capability; (ii) depolyploidization by an amitotic processes called neosis, which involves budding and bursting, similar to prokaryotes and unicellular eukaryotes, to generate tumor initiating cells with cancer stem cell-like properties; (iii) horizontal transmission of sub-genomic material via cytoplasmic tunnels, conferring the recipient (small-sized) cells with cancer stem cell-like properties; and (iv) secretion of factors that support tumor growth and progression (for details, please see [29,30,47,48]).

**Figure 4:**
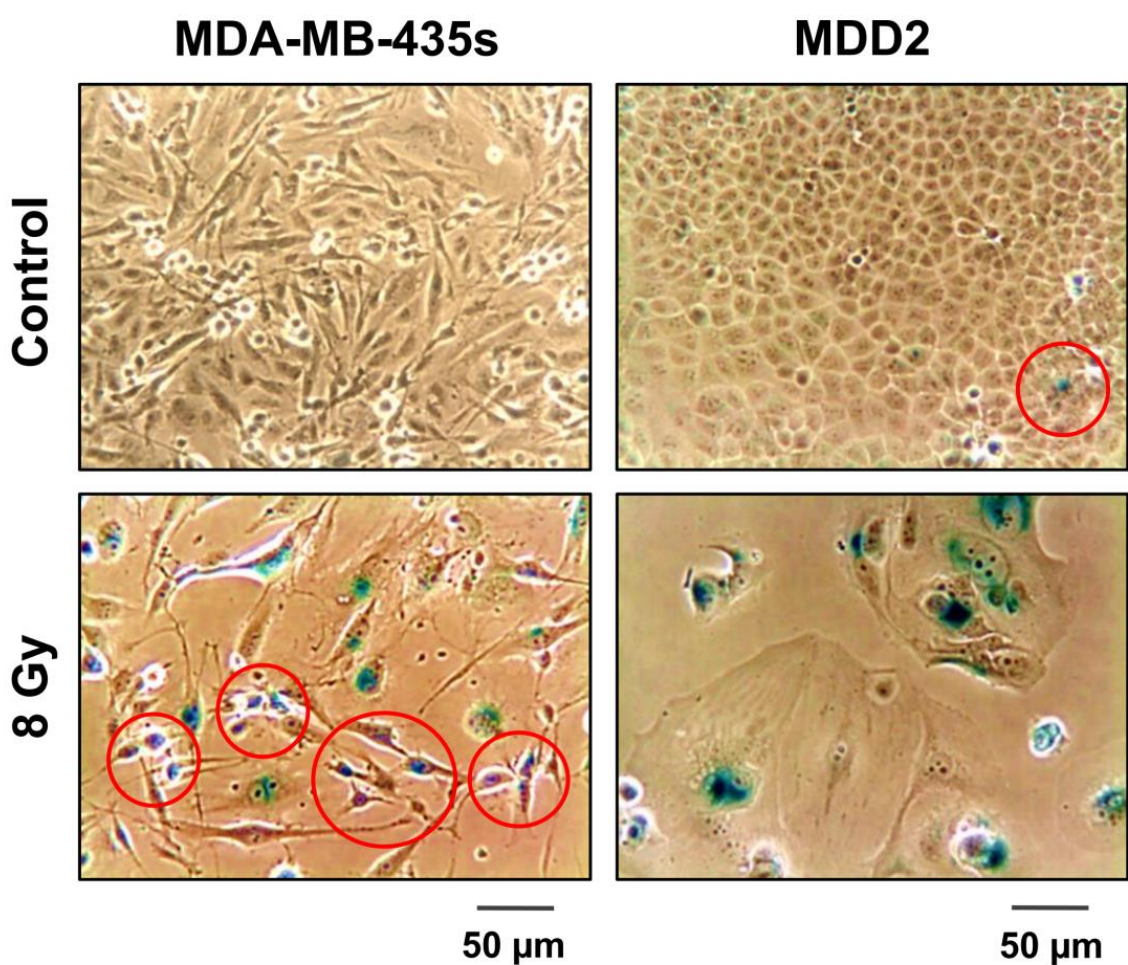
Phase-contrast microscopy images showing features of senescence in the indicated breast cancer cell lines. Cells were exposed to ionizing radiation (8 Gy) or sham-irradiated (control), incubated for seven days, and evaluated for morphology and positive (blue) staining in the senescence-associated β-galactosidase (SA β-Gal) assay. Some regions containing “small-sized” SA β-Gal-positive cells are marked. Reproduced from Mirzayans et al. [51].

The development of giant cells following anticancer treatment is not always associated with senescence. In fact, SA β-Gal-positive and -negative giant cells can be present in the same culture of a cancer cell line [20]. On the other hand, triggering cancer cell senescence following radio/chemotherapy exposure is not always associated with the presence of a highly enlarged nucleus, multiple nuclei, or multiple micronuclei. For example, we have observed that SKNSH neuroblastoma cells have a high propensity to undergo senescence following exposure to ionizing radiation [49] and that the majority of these (>90%) remain in this dormant state for long times (up to three weeks) post-irradiation without exhibiting manifestations of polyploidy, multinucleation, or micronucleation (unpublished observations). It is also important to note that cancer cell dormancy is not always associated with highly enlarged/flatted morphology. Such “small-sized” dormant cells include drug-tolerant persister (DTP) cancer cells [50], as well as SA β-Gal-positive cells within some cancer cell lines, including the MDA-MB-435s breast carcinoma cell line ([51]; also see Figure 4).

It is important to note that most cancer cell lines that were used in senescence-related studies over a decade ago were subsequently shown to enter the polyploidy-stemness route. These include the colon carcinoma cell lines HCT116 and SW480 [52] and the breast carcinoma cell lines MCF7 and MDA-MB-231 [52,53] after treatment with chemotherapeutic drugs.

### 2.3. Contributions of Our Group to the Understanding of the Creation and Fate of PGCCs following Anticancer Treatment

**Figure 5:**
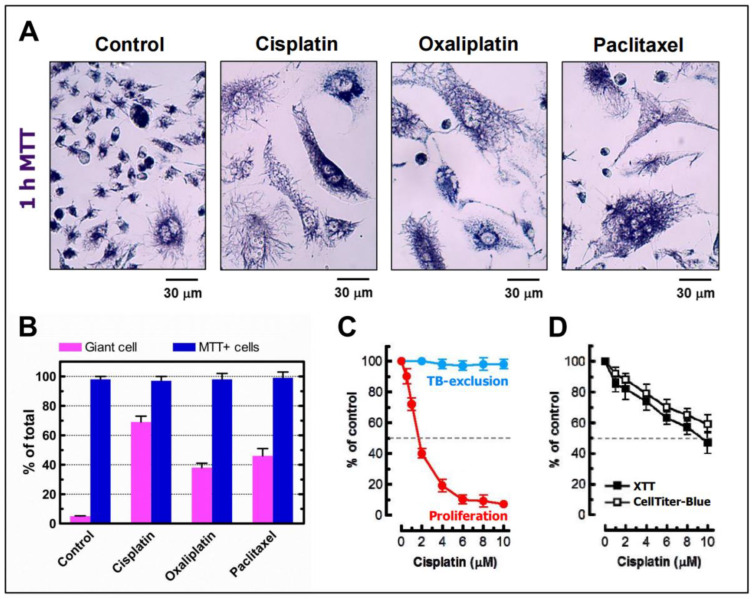
(**A**) Bright-field microscopy images showing the ability of MDA-MB-231 cells to convert the MTT reagent to its formazan metabolite (dark granules and crystals) before (control) and after incubation with cisplatin (10 µM), oxaliplatin (10 µM), or paclitaxel (20 nM) for 3 days. Images were acquired after incubation of cells with MTT for ~1 h. (**B**) Percentages of polyploid/senescent giant cells and MTT-positive cells in cultures of the MDA-MB-231 cell line before (control) and after treatment with cisplatin (10 μM), oxaliplatin (10 μM), or paclitaxel (20 nM) for 3 days. Only adherent cells were evaluated. Bars, standard error (SE). (**C**) Effect of cisplatin treatment (3 days) on the extent of cell proliferation (determined by the direct cell counting assay) and cell membrane integrity (determined by the trypan blue-exclusion assay). Bars, SE. TB, trypan blue. (**D**) Response of MDA-MB-231 cells to cisplatin (3-day incubation with the indicated concentrations), evaluated by the 96-well plate XTT (solid squares) and CellTiter-Blue (open squares) “viability” assays. These images and data are reproduced from Mirzayans et al. [32,55].

Our group has focused largely on determining the contribution of PGCCs to radiosensitivity [54] and chemosensitivity [32,55] as measured by cell proliferation (colony formation and/or direct cell counting) assays, multiwell plate colorimetric/fluorometric assays, and various single-cell assays. We have shown that the responses measured by these assays in solid tumor-derived cell lines predominantly reflect proliferation arrest (dormancy) through the creation of PGCCs, irrespective of the status of p53-p21 signaling. Importantly, we have shown that cancer cells (including PGCCs) that remain adherent to the culture dish at any time point (up to 3 weeks) after exposure to clinically relevant doses of anticancer agents remain viable and metabolically active. This was evaluated by a simple assay that we have optimized [32,54,55,56], which is based on the ability of individual cells to convert the tetrazolium salt 3-(4,5-dimethylthiazol-2-yl)-2,5-diphenyl-tetrazolium bromide (MTT) to its water-insoluble formazan derivative (the so-called “single-cell MTT” assay). Our data obtained with chemotherapy-treated MDA-MB-231 breast cancer cells is reproduced in Figure 5. It shows, for example, that a 3-day exposure to 10 µM cisplatin results in an almost total proliferation block, which largely reflects the formation of PGCCs that exhibit the ability to metabolize MTT.

## 3. Important Considerations When Assessing Cancer Cell Radiosensitivity and Chemosensitivity? What Does “Sensitivity” Actually Refer to?

Since the 1990′s, our group has contributed to the understanding of the roles played by ATM, p53, WIP1, p21, and p16^INK4a^ (p16) in the DNA damage response (reviewed in [51,57,58,59,60]). Those early days of the DNA damage response era led to a number of assumptions that have become almost “undisputable facts,” and yet have not been supported by solid experimental data, or indeed have proven to be untenable. Some of these “hypotheses,” together with discoveries (both old and new) that need to be taken into consideration when assessing cancer cell response to therapeutic agents, are briefly discussed below.

### 3.1. Significance of p53-p21-WIP1 signaling in Suppressing Cancer Cell Death and Triggering (Reversible) Senescence

In numerous (thousands of) articles, wild-type p53 is assumed to be pro-apoptotic. This is somewhat surprising because by 2008 it was already well established that under physiological conditions (e.g., absence of ectopic gene expression) wild-type p53 in fact suppresses apoptotic cell death in certain cell types (e.g., solid tumor-derived cell lines) by regulating approximately forty anti-apoptotic proteins, including p21 and WIP1 [61]. As expected, the list of p53-regulated pro-survival factors has grown over the years (see, e.g., [58,62,63]). Thus, although ectopic expression of wild-type p53 can induce some aspects of apoptosis, activation of p53-p21-WIP1 signaling serves primarily to suppress cell death and instead triggers (prolonged but reversible) proliferation arrest through premature senescence.

The reversibility of cancer cell senescence following chemotherapeutic exposure was established in the early 2000’s and was suggested to be associated with the absence of p16 function (see, e.g., landmark studies reported by Igor Roninson’s group, reviewed in [64]). Paradoxically, there is now evidence that cell cycle re-entry (reversal of the proliferation arrested state of senescent cancer cells) can be accelerated by ectopic expression of caspase 3 or treatment with apoptosis-triggering anticancer drugs such as camptothecin and the BCL2 inhibitor ABT-737, and that this re-entry produces aggressive variants [65]. (The cancer cell lines used in the latter study are known to be p16-suppressed through epigenetic gene silencing, although this was not mentioned by the authors.)

### 3.2. Pro-Survival Properties of Cancer Cells Triggered to Undergo Apoptosis

**Figure 6:**
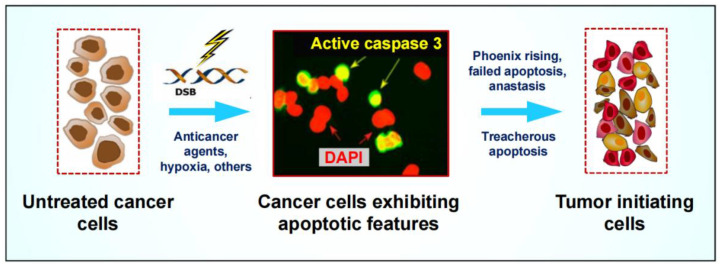
Cartoon illustrating the dark side of apoptosis. Cancer cells with molecular, biochemical, and morphological features of apoptosis are capable of promoting tumor repopulation via different routes, including: (i) secretion of pro-survival factors that is regulated by caspase 3 and involves various signaling pathways, including JNK (c-Jun N-terminal kinase); and (ii) the ability to return from the brink of apoptotic death, resulting in the emergence of progeny with increased numbers of micronuclei and chromosomal abnormalities that can lead to increased aneuploidy, a driving force of aggressive cancer (reviewed in [17]). These various oncogenic functions associated with “dying” (apoptotic) cancer cells include phoenix rising, failed apoptosis, and anastasis. “Treacherous apoptosis” refers to regions within a tumor that are enriched with caspase 3-positive cells (see text for details).

In 2013, Malathy Shekhar published a comprehensive book chapter entitled “The Dark Side of Apoptosis” in which she discussed accumulating clinical evidence for the paradoxical role of apoptosis in tumor progression [66]. Since then, the oncogenic functions of caspase 3 [17,67,68], together with the ability of cancer cells to return from the brink of apoptotic and other modes of cell death through anastasis [16,17], have all been well established and extensively reviewed (also see Figure 6). Recently, Khatib et al. [69] have provided further evidence supporting the pro-survival features of apoptotic cancer cells. By analyzing a large number of hepatocarcinoma tumor samples by a variety of single-cell assays, these authors identified densely populated caspase 3-positive regions (apoptosis islands) within an individual tumor, and further demonstrated that higher levels of apoptosis led to increased therapy resistance, reflecting the therapeutic implications of intratumor heterogeneity. In an Editorial entitled “Treacherous apoptosis…” Dhanasekaran summarized these discoveries and concluded that this phenomenon (pro-survival apoptotic islands) provides an explanation for the observation that tumors with a high apoptotic index tend to have a poor prognosis [12].

### 3.3. Danger of Relying on High Content Multiwell Plate Assays for Cancer Cell “Lethality” Assessment

In numerous articles it is assumed that high content multiwell plate cell “viability” or “cytotoxicity” assays can be used to assess cancer cell death. However, when performed with proliferating cultures, these assays are highly non-specific. In 2017, Eastman [15] provided detailed evaluations of treatment-induced responses measured by such assays, and concluded that these assays primarily measure proliferation arrest rather than loss of cell viability (which is often misinterpreted to reflect cell death). As we have recently pointed out [10], various responses (mostly long-term pro-survival) contribute to the IC_50_ values (drug concentrations resulting in 50% inhibitory effect) when measured by multiwell plate colorimetric (e.g., tetrazolium-based; crystal violet-based) and fluorometric (resazurin-based, such as CellTiter-Glo) assays. These responses include: (i) a decrease in the metabolic activity of individual cells (this effect will not influence the IC_50_ values measured by the crystal violet staining assay); (ii) transient cell cycle checkpoint activation, which promotes survival by facilitating DNA repair; (iii) short-term proliferation arrest reflecting anastasis (return journey from the brink of death) that might ultimately lead to the emergence of aggressive variants; (iv) short-term proliferation arrest reflecting transient loss of cell membrane integrity (e.g., as a result of chemotherapy exposure, commonly detected by large dye uptake assays) which can be rapidly restored; (iv) long-term proliferation arrest (dormancy via creation of PGCCs, which includes senescence) that may or may not be reversible, but is often not associated with loss of viability; and (v) bona fide loss of viability (cell death). In short, to assume that the effect measured by such colorimetric/fluorometric assays reflects cancer cell “lethality” can be highly misleading. Furthermore, the emerging complexity of the cellular response to therapeutic agents underscores the significance of single-cell (versus population-averaged) observation methods for the assessment of cancer cell viability and metabolic activity (also see Section 3.5).

### 3.4. Danger of Relying on the Clonogenic “Survival” Assay for Assessment of Cancer Cell Death

As recently pointed out by Brix et al. [70], “the clonogenic assay is widely used to test reproductive cell survival in vitro. Developed already in the 1950s by Puck and Marcus, it has proven a powerful methodology to assess sensitivity towards radiotherapy, chemotherapy, as well as molecularly targeted therapy, and undoubtedly represents the in vitro gold standard in this regard.” Indeed, this so-called clonogenic “survival” assay, first used by Puck and Marcus in 1956 [71] to determine the response of HeLa cervical carcinoma cells to ionizing radiation, has been widely used for decades as the key biological indicator of cancer cell death by radiation/cancer biologists in general, and by the synthetic “lethality” community in particular (reviewed in, e.g., [56]).

**Figure 7:**
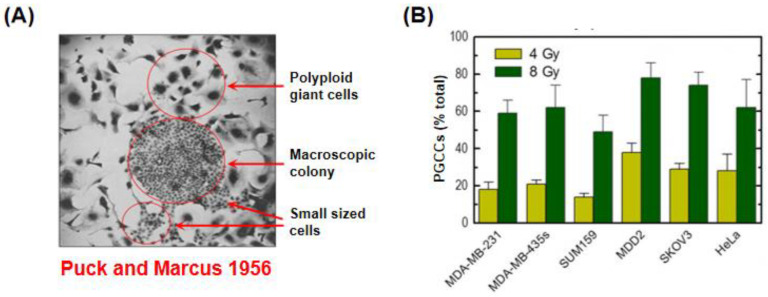
(**A**) A phase-contrast microscopy image reproduced from the original work of Puck and Marcus that was published in 1956 [71], reporting the effect of ionizing radiation (9 Gy) on the colony-forming ability of HeLa cell cultures. The image shows remarkable (~10 times) size differences between proliferating (colony forming) cells and proliferation arrested polyploid giant cells. (**B**) Data obtained by us [54] with HeLa and the indicated cell lines that were exposed to ionizing radiation and incubated for 3 days.

Critically, a seminal discovery of Puck and Marcus that was also reported in their 1956 article [71] was not mentioned by Brix et al. [70], and has previously been overlooked by us (reviewed in [20]) and others (too many to cite). Namely, detailed evaluation of HeLa cells that failed to produce macroscopic colonies (aggregates of at least 50 cells) within ~10 days after irradiation showed two important observations regarding the fate of HeLa cells that were ostensibly “killed” following radiation exposure. First, a large proportion of cells that lost the ability to form a colony after exposure to any dose of radiation gave rise to one or more giant cells with extremely enlarged morphology and nuclear content; the phase-contrast microscopy image showing the morphology of giant cells and small-sized (colony-forming) HeLa cells presented by Puck and Marcus is reproduced in Figure 7A. (Our data obtained with HeLa and other solid cancer-derived cell lines are presented in Figure 7B.) Second, these giant cells metabolized at a high rate (as judged by their ability to change the pH of the growth medium) and could be maintained in the metabolically active state for long times (e.g., three weeks) if the medium was regularly replenished. As we noted previously [20], these critical observations prompted the development of the feeder layer colony formation assay, in which heavily irradiated feeder (giant) cells are seeded in culture dishes at a relatively high density to promote the proliferation of test cells via secreted factors. These remarkable observations reported some 67 years ago have raised a key question that unfortunately most of us in the field have overlooked: if the majority of 9 Gy-irradiated cancer cells remain adherent to the culture dish, retain cell viability, and secrete a myriad of factors for long times (weeks) post-irradiation, how can they be considered to be dead (as still appears to be the case in most preclinical cancer therapy-related publications)?

Take home message: When the “gold standard” colony formation and other preclinical assays (e.g., high content cell “viability”) are used to evaluate radiosensitivity and chemosensitivity, for some cell types (notably, solid tumor-derived cell lines), this sensitivity predominantly reflects the treatment-induced conversion of dangerous (proliferating) cancer cells to potentially even more dangerous (dormant) tumor repopulating cells that exhibit various manifestations of genome chaos (e.g., polyploidy, multinucleation, micronucleation, senescence, apoptosis-associated DNA strand breaks), rather than dead cancer cells.

### 3.5. Single Cell Biology: A Step towards Generating Clinically Relevant Information

In 2014, Robert Weinberg published a Leading Edge Essay in *Cells* [72] in which he discussed the danger of merely relying on information-generating approaches to cancer research. He wrote, “…we have come full circle, beginning in a period when vast amounts of cancer research data yielded little insight into underlying mechanisms to a period (1980–2000) when a flurry of molecular and genetic research gave hope that cancer really could be understood through simple and logical reductionist thinking, and finally to our current dilemma. Once again, we can’t really assimilate and interpret most of the data that we accumulate.” Unfortunately, this information generating approach continues to dominate the various aspects of cancer research (too many to cite).

Unlike the majority of cancer researchers who rely heavily on conventional anticancer assays, a handful of scientists, our group included (e.g., [56]), have published research/review articles to highlight the importance of single cell biology in obtaining preclinical information of clinical relevance. Zaitceva et al. [16], for example, published a comprehensive review entitled “Anastasis: Return Journey from Cell Death” in which they concluded that “…the live single cell analysis is the most precise way to determine the real efficacy of anti-cancer treatment and allow the prediction of relapse because of surviving subpopulations. Unfortunately, single-cell assays are more complicated methods compared to cell-based population assays and not very affordable, which discourages their widespread use as preclinical tests for the evaluation of treatment cytotoxicity. Therefore, as cells recovered from death are more aggressive and genomically unstable, it is extremely important to distinguish dead from dying cells.”

The single-cell MTT assay that we have optimized is particularly useful in distinguishing dead cancer cells from dormant cancer cells (i.e., cells that remain viable and metabolically active but fail to generate macroscopic colonies) and dying (apoptotic) cancer cells that have the ability to return from the brink of death [56]. It simply involves adding the MTT solution to the culture medium and incubating the cells for ~1 h. Viable and metabolically active cells, irrespective of their morphology and proliferation state, rapidly convert MTT to its water insoluble purple formazan metabolite, which can be visualized as purple intracellular granules and crystals under a light microscope (see, e.g., Figure 5A).

## 4. Possible Reasons Why PGCCs and Their Tumor Repopulating Properties Continue to Be Overlooked in Most Preclinical Anticancer Studies

### 4.1. Misleading Assumption That PGCCs Represent Dead or Dying Cells That Will Be Eventually Eliminated via Apoptosis and Other Means

Giant cancer cells with massive nuclear contents have been described by physicians and scientists since over a century ago [73]. However, as recently pointed out by Pienta et al. [73], the “majority of the cancer research and treatment development communities have disregarded these cells as irreversibly senescent or destined for mitotic catastrophe and death. A small number of pioneering scientists, including Erenpreisa, Cragg, Illidge, Liu, Walen…(and others)…have now made it clear that these cells…(PGCCs)…are important mediators of tumorigenesis, metastasis, and therapeutic resistance.” In addition to these scientists, and Pienta and Amend for their recent remarkable contributions to this field ([36,48,73]), we wish to acknowledge Henry Heng [27,28] for bringing to our attention, about a decade ago, the importance of genome chaos (various manifestations of “mitotic catastrophe”) in therapy resistance and disease recurrence. 

### 4.2. Misleading/Inappropriate Preclinical Assays?

It is also possible that PGCCs continue to be ignored by the majority of cancer researchers because conventional preclinical assays that are widely used to identify novel anticancer agents and therapeutic strategies are not designed to incorporate the heterogeneity and complexity that exists within a tumor ([10]). Furthermore, the time required between therapeutic exposure and the emergence of tumor repopulating progeny of cancer cells that are triggered to undergo dormancy through polyploidy/senescence is much longer (weeks to months) than the time span of multiwell plate cell “viability” (e.g., MTT, CellTiter-Glo, etc.), colony formation, and other widely used preclinical anticancer assays for cell “killing” (reviewed in [10]; also see Figure 3), such that these assays would not be informative for these longer-term responses.

### 4.3. Dishonesty in Data Reporting?

Some of the caveats regarding progress in cancer research that were pointed out by the Nobel Prize Laureate William Kaelin [74,75] and others (e.g., [76]) may also help to explain why PGCCs are widely overlooked (reviewed in [17]). These include the pressure to “publish or perish” that may result in exaggerations about the significance or certainty of research findings, and sometimes may even lead to publishing massaged or falsified results. This might appear to be a harsh statement, but major journals do retract exaggerated/falsified papers that are published in various fields, including DNA repair (e.g., [77]), p53 signaling (e.g., [78]), and synthetic “lethality” (e.g., [79,80]). In fact, some journals have introduced several data screening checks before accepting manuscripts for publication in an attempt to reduce the number of post-publication retractions (e.g., [81]). The increasing frequency of dishonesty in cancer therapy-related manuscripts was highlighted in an Editorial entitled “Figure errors, sloppy science, and fraud: keeping eyes on your data” that was published in *Journal of Clinical Investigations* in 2019 [81]. The authors stated that “on the journal side, we are limited to catching obvious errors after they are committed. The scientific community as a whole needs to be steadfast in guarding against unreliable data at all stages of planning, acquiring, interpreting, and publishing data.”

We performed online searches to see if there are any updates regarding retracted papers since our last review on this subject [17]. Shockingly, over FIFTY p53/cancer-related articles have been retracted since 2021! We also came across a blog on “retraction watch” [82] which highlights five major papers retracted from a reputable laboratory. In this blog, the important question is raised: has anyone, or organization, “started to audit meta-analyses, systematic reviews, practice guidelines, etc—to determine the impact of these retractions?” This is a profound question, which illustrates the negative impact of sloppiness in biomedical research. We have a similar concern about the majority (thousands) of authors who have published cancer cell “lethality” articles by merely relying on measuring cell “viability” (by high content multiwell plate assays), some ambiguous manifestations of apoptosis (e.g., caspase 3 activation), and/or proliferation arrest (“mitotic catastrophe”) as markers of cancer cell death. Like the retracted papers, how are these highly biased articles going to impact “meta-analysis, systematic reviews, practice guidelines, etc?”

## 5. Relevance to the Future Direction of Precision Oncology: A Personal Perspective

The transition from the one-size-fits-all approach to the treatment of solid tumors to patient-individualized precision oncology based on the molecular profiling of their tumors has been regarded as a powerful and compelling strategy. Unfortunately, as discussed by us [17] and other groups (e.g., [83,84,85]), progress in this area has been rather slow. Here we have discussed some of the reasons for this lack of progress, such as the failure of many researchers to recognize that achieving the “Holy Grail” of cancer cell death using conventional and experimental therapies alike remains largely unfulfilled. In fact, rather than promoting cancer cell “death”, these therapies commonly drive cancer cells into a state of dormancy that can involve potentially aggressive and treatment-resistant entities such as PGCCs, which have been largely ignored by the cancer research community. Some questions that will need to be answered if this field is to start moving forward include:

● Classical DNA-damaging cancer therapeutics (e.g., ionizing radiation, cisplatin) have been shown to induce significant PGCC formation (reviewed in [20]). Do more recent cancer mutation-targeted strategies such as exploiting synthetic–“lethal” partnerships also cause the generation of PGCCs?
● Can we develop reliable and high throughput imaging-based versions of the currently cumbersome and expertise-dependent assays for entities such as PGCCs that will be accepted/taken up by the scientific community such that screening these responses to therapy in tissue culture can be done in a time- and cost-effective manner?
● With such assays in hand, can we identify drugs/combinations (with or without radiation) that either circumvent the generation of these treacherous PGCCs or trigger their demise?
● Given that PGCCs are highly atypical in many regards, such as their size, shape, and ploidy, can we devise strategies that will harness the full power of the immune system to eradicate these potentially harmful aberrant cells?

In addition to addressing these questions, to realize the monumental goal of combating cancer, it is important to take into account not only the mutational basis of the intrinsic/acquired therapy resistance of cancer cells, such as the many examples highlighted in most reviews on targeted therapies (e.g., [81,82,83]) as well as the dark side of apoptosis (e.g., [12,16]), but also the non-mutational events discussed previously [10,17], which include the creation of PGCCs via cell fusion (e.g., [86,87,88,89]).

## 6. Conclusions

We have previously discussed the multifactorial nature of cancer cell resistance to DNA-damaging therapeutic agents, far beyond the simplistic model of “repair and survive or die through apoptosis or other regulated cell death pathways” [10]. In the current review, we have provided an update on PGCCs, a root cause of therapy resistance and disease recurrence, with an emphasis on the prognostic value of PGCCs across different cancer types, and possible reasons why these tumor repopulating giants continue to be overlooked by most cancer researchers.

Our current understanding of the (literally) mind-numbing complexity and heterogeneity that exists within a solid tumor (see, e.g., Figure 1) should be considered a huge step forward in terms of the metaphor of the “war on cancer” because, at the very least, we have learned a great deal as to who the various subsets of “enemies” are. In this context, it is important to note that one such “enemy” does not reside in the tumor (Figure 1), but rather pertains to experimental design, with a widespread use of highly simplistic and non-specific preclinical assays that, in our opinion, has derailed cancer research for ~50 years, as discussed above.

Food for thought: As we suggested previously [17], “…perhaps efforts of cancer researchers should be primarily directed towards prevention, rather than employing the same misleading preclinical assays and wishy-washy interpretations (to quote William Kaelin [75]) with “novel” anticancer drugs and catchy names for treatment strategies (e.g., “synthetic lethality”) to expect different outcomes...” There might be an exception to this conclusion with potential clinical relevance. Given the various mechanisms by which different subsets of cancer cells within an individual tumor escape death post-therapy, together with challenges encountered in cancer immunotherapy (e.g., cytokine “storms” that can cause severe toxicity and even death [90]), modern therapies should be largely ineffective in patients with a solid tumor. But there are cases where even conventional radio/chemotherapy seems to result in long-term (over 5 years) remission, and may even lead to cancer cure. This raises a fundamental question. What are the reasons that some cancer patients do well even after undertaking conventional therapies? Is it possible that their immune system is capable of destroying tumor repopulating “outliers” within a tumor (e.g., PGCCs, oncogenic caspase 3-acivated cells) before they will have the opportunity to promote tumor progression (through secretory factors) and to give rise to tumor repopulating progeny, or perhaps such “outliers” are not present at significant frequencies in the tumors of these patients (before and after therapy) in the first place? In other words, what fundamental factors underlie inter-tumor heterogeneity (heterogeneity between patients with the same type/stage of cancer) in terms of therapy response? We wouldn’t pretend to know the answer, and assume that addressing it will involve decades of work and investment. But it may be worth the effort!

## Figures and Tables

**Table 1:** Prognostic value of polyploid/senescent cancer cells.

Author	Date	Cancer Type	No of Patients	Outcome
Wang et al. [22]	2012	Non-small-cell lung cancer	18	Patients expressing markers of senescence following neoadjuvant therapy had a significantly worse prognosis than patients who did not express these markers.
Qu et al. [37]	2013	Glioma	76	The number of PGCCs increased with the grade of tumors.
Lv et al. [42]	2014	Serous ovarian cancer	80	The presence of PGCCs in the primary tumor correlated with metastasis.
Fei et al. [40]	2015	Primary breast tumors, lymph node metastases, and benign tissue	167	The number of PGCCs was the highest in patients with lymph node metastases.
Gerashchenko et al. [41]	2016	Breast cancer	30	Tumors with a higher proportion of PGCCs showed a poorer response to neoadjuvant chemotherapy.
Zhang et al. [43]	2017	Colon cancer	169	The presence of PGCCs with budding increased as tumors became more dedifferentiated.
Liu et al. [38]	2018	Anorectal melanoma	47	The proportion of PGCCs increased with tumor size.
Alharbi et al. [46]	2018	Prostate cancer	30	Pleomorphic giant cells were present in all 30 patients with a rare variant of prostate cancer.
Mannan et al. [45]	2020	Prostate cancer	5	Multiple cells with highly irregular polylobulated nuclei or multiple pleomorphic nuclei were present in autopsy samples of patients who had failed multiple lines of therapy.
Liu et al. [39]	2021	Laryngeal cancer	102	High numbers of PGCCs correlated with poor prognosis.
Trabzonlu et al. [36]	2023	Prostate cancer	209	PGCCs were significant prognostic factors for metastasis in patients who underwent radical prostatectomy with curative intent to treat their presumed localized cancer.
